# Beyond ‘Baby Brain’: Memory and Sleep Health During Pregnancy—A Biopsychosocial Perspective

**DOI:** 10.3390/brainsci16080821

**Published:** 2026-07-31

**Authors:** Elisabetta Baldi, Johanna Gfüllner, Debora Meneo, Silvia Cerolini, Kerstin Hödlmoser, Chiara Baglioni

**Affiliations:** 1 Department of Human Sciences, Guglielmo Marconi University, 00193 Rome, Italy; e.baldi@unimarconi.it (E.B.); d.meneo@unimarconi.it (D.M.); s.cerolini@unimarconi.it (S.C.); 2 Laboratory for Sleep, Cognition and Consciousness Research, Centre for Cognitive Neuroscience (CCNS), Department of Psychology, University of Salzburg, 5020 Salzburg, Austria; johanna.gfuellner@plus.ac.at (J.G.); kerstin.hoedlmoser@plus.ac.at (K.H.); 3 Department of Psychiatry and Psychotherapy, Medical Center—University of Freiburg, Faculty of Medicine, University of Freiburg, 79104 Freiburg, Germany

**Keywords:** pregnancy, memory, sleep health, biological factors, psychosocial factors

## Abstract

Across the course of pregnancy, women undergo profound biological, psychological, and social changes, often accompanied by subjective memory complaints. However, evidence for objective memory problems remains inconsistent, revealing a gap between subjective and objective impairment. This narrative review aimed to (a) provide an overview of memory difficulties during pregnancy, with particular attention to the potential biological and psychosocial mechanisms underlying them, and (b) examine sleep health as a specific and potentially modifiable factor within this framework, adopting a multidimensional perspective. Findings indicate that pregnant women frequently report subjective memory difficulties, whereas objective impairment is generally subtle, domain-specific, and remains within normative ranges. Biological evidence suggests substantial hormonal fluctuations and dynamic brain reorganisation during pregnancy; however, consistent associations with memory decline have not been established, and some studies point to adaptive neuroplastic processes. At the same time, societal expectations and environmental factors may influence subjective memory functioning, contributing to the discrepancy between subjective and objective findings. Sleep is consistently compromised during pregnancy and plays a key role in memory processes. However, evidence linking sleep and memory during pregnancy is limited and heterogeneous, with predominantly correlational findings across different dimensions of sleep health. These results support the need for a biopsychosocial framework that incorporates a multidimensional conceptualisation of sleep and for overcoming simplified views of pregnancy psychophysiological processes. Future research should adopt longitudinal and multimodal approaches to clarify the role of sleep health and its interaction with biological and psychosocial factors, ultimately informing preventive strategies and more comprehensive models of maternal memory functioning.

## 1. Introduction

Pregnancy is a sensitive life period with respect to women’s functioning across multiple domains. Although pregnancy is a natural process, it involves profound biological, psychological, and social changes that require women to adapt to new physical conditions, roles, and responsibilities [1], potentially exposing them to a range of challenges. During pregnancy, women may report subjective memory complaints and are often perceived as forgetful, stressed, anxious, and cognitively impaired [2,3,4,5,6]. Perceived (i.e., subjective) memory deficits are among the most reported complaints during pregnancy [2], although these subjective reports may not necessarily reflect objective changes in memory performance [5]. Importantly, these perceptions emerge within a social context characterised by pervasive maternity-related stereotypes and prejudices, which portray pregnant women as warm but less competent [6]. These sociocultural stereotypes may shape women’s self-perceptions and reports of memory changes [4], contributing to the well-known phenomenon commonly referred to as “baby brain” [2]. While self-report studies often suggest substantial memory difficulties, objective findings remain mixed [7], leaving open the question of whether perceived memory changes primarily reflect biological mechanisms or are partly driven by societal expectations.

Identifying basic biological and psychosocial processes that may contribute to memory complaints during pregnancy is, therefore, essential for moving beyond stereotypical interpretations. One such process, crucial for both mental health and cognitive functioning, is sleep. Sleep has been consistently associated with memory [8,9,10,11,12,13,14,15,16]. Among women, sleep is often impaired during sensitive life stages such as pregnancy and the postpartum period [17]. According to the National Sleep Foundation, up to 84% of women report poor sleep quality during the peripartum period [18]. Pregnant women have a higher risk of sleep disorders and poorer sleep health than non-pregnant women, including poor sleep satisfaction [19], insufficient or excessive sleep duration [20], and sleep timing variability [21].

Rather than restricting investigation to clinically disturbed sleep, recent research has increasingly adopted a positive, multidimensional, and health-oriented conceptualisation of sleep, which provides a more comprehensive framework for identifying early vulnerabilities and potential risk chains [22,23]. According to Buysse [22], “sleep health” can be defined as a continuum, ranging from optimal to poor sleep health, and includes five different dimensions: sleep satisfaction, daytime alertness (or sleepiness), sleep timing and regularity, sleep efficiency, and sleep duration. Alterations in each one of these dimensions are associated with poor health outcomes even in non-clinical populations [22]. A sixth dimension—pre-sleep behaviours—has been introduced more recently in the paediatric population [24], and it has been proven to be relevant for adults as well [25,26,27]. The sleep health framework provides a useful approach for integrating existing research, identifying gaps in the literature, and recognising promising future directions for research and clinical interventions [23]. Furthermore, this broader conceptualisation of sleep as a multidimensional aspect of health conforms to the World Health Organisation’s view, which describes “health” as a “state of complete physical, mental and social well-being and not merely the absence of disease or infirmity” [28]. Overall, peripartum is characterised by profound biopsychosocial changes, which could compromise all dimensions of sleep health. Although there is extensive evidence that sleep health is frequently impaired during pregnancy and that women commonly report subjective memory difficulties, as well as evidence that sleep plays a key role in memory processes, very few studies have examined the relationship between specific dimensions of sleep health and memory during pregnancy. Consequently, it remains unclear whether poor sleep health contributes to the memory complaints often reported in this population. Addressing this gap is essential for better understanding maternal memory functioning and for guiding clinical and public health initiatives.

Therefore, this narrative review aims to: (a) provide an overview of memory difficulties observed during pregnancy, considering the potential biological and psychosocial mechanisms that may underlie these issues; and (b) examine sleep health as a potentially modifiable underlying factor, by summarising the existing literature on sleep health, memory, and their interactions during gestation. Unlike previous reviews, which primarily synthesised evidence on pregnancy-related cognitive changes through quantitative (meta-analytic) or systematic approaches [5,7,29], this narrative review adopts a biopsychosocial framework to provide a qualitative synthesis of how dynamic interactions among biological and psychosocial processes, particularly sleep health, may contribute to subjective and objective memory functioning during pregnancy. Within this perspective, memory functioning during pregnancy is understood not as the consequence of isolated biological changes, but as an outcome emerging from the interplay between physiological adaptations, individual experiences, and social influences. By doing so, this review seeks to synthesise current evidence, identify gaps in the literature, and highlight the relevance of investigating the sleep health–memory relationship during pregnancy for both research and healthcare services. Ultimately, a better understanding of the mechanisms linking sleep health and memory may help identify modifiable targets for future preventive interventions and inform clinical care for women reporting memory complaints during pregnancy.

## 2. Methods

The literature included in this narrative review was identified through two complementary approaches.

To identify studies investigating the association between sleep health and memory during pregnancy, a structured literature search was conducted in PubMed, PsycINFO, and Scopus, with Google Scholar additionally used to identify potentially relevant publications not captured through database searches. The search was performed in September 2025 using combinations of keywords related to pregnancy (e.g., “pregnan*”, “prenatal”, “antenatal”, “perinatal”), memory (e.g., “memory”, “mnestic”, “cognit*”), and sleep (“sleep”). Eligible studies included original cross-sectional or longitudinal research examining the association between at least one dimension of sleep health and any aspect of memory functioning in healthy adult pregnant women. Studies focusing exclusively on participants with major somatic or psychiatric disorders were excluded to minimise potential confounding effects on sleep and cognitive functioning. In case–control studies comparing pregnant and non-pregnant women, only data from the pregnant participants were considered, as the aim of this review was to characterise the association between sleep and memory within the pregnant population rather than to examine differences between pregnant and non-pregnant women. Reviews, case reports, and intervention studies involving experimental sleep manipulation (e.g., randomised controlled trials) were excluded.

To contextualise these findings, additional targeted literature searches were conducted primarily in the same electronic databases between September 2025 and February 2026. Searches were performed progressively according to the specific topic being addressed. For the section on pregnancy-related memory functioning, searches combined pregnancy-related terms with keywords related to memory and cognition and were informed by previous reviews in the field, with relevant primary studies identified through reference list screening where appropriate. For the section on biological mechanisms, searches additionally included terms related to hormonal changes (e.g., “hormon*”, “progesterone”, “oestradiol”, etc.) and to brain structure, brain function, and neuroimaging techniques (e.g., “fMRI”, “functional connectivity”, “structural changes”, “brain plasticity”, etc.). For psychosocial mechanisms, searches combined pregnancy-related terms with keywords referring to psychological factors (e.g., “concerns”, “worry”, “emotional distress”, “neuroticism”, etc.) and social factors (e.g., “social support”, “role transition”, “work demands”, “stereotypes”, etc.). These searches were also informed by relevant reviews and supplemented by screening the reference lists of eligible publications to identify additional studies.

Furthermore, beyond the searches conducted up to February 2026, additional targeted literature searches were undertaken throughout the development of the manuscript whenever discussions among the co-authors identified the need to expand, clarify, or update specific sections and topics.

## 3. Memory During Pregnancy and Its Underlying Factors

Memory during pregnancy has received considerable scientific attention, largely due to the high prevalence of self-reported memory changes among expectant mothers. Over the past few decades, research on pregnancy-related cognitive changes has increased substantially [29]. Despite this growing body of literature, findings remain inconclusive. “Pregnancy brain” or “baby brain” are widely used stereotypical colloquial terms describing perceived memory lapses, forgetfulness, and absentmindedness reported by pregnant women [29,30]. While 50–80% of pregnant women report increased forgetfulness and a perceived decline in memory during pregnancy [2,31], objective assessments of memory performance have yielded mixed results, with some studies reporting subtle impairments and others finding no significant differences compared to non-pregnant controls [29]. This discrepancy highlights the importance of: (a) distinguishing between subjective experiences of memory difficulties and objectively measured memory performance; (b) understanding the underlying biological and psychosocial processes that may explain memory impairment in some pregnant women.

Pregnancy is characterised by profound physiological and biological changes, alongside psychosocial and environmental ones, that may influence self-reported memory problems. Fluctuations in hormonal systems affecting memory and alertness functions have been proposed as potential mechanisms underlying pregnancy-related cognitive changes [32,33,34,35]. However, the extent to which hormonal and other biological processes contribute to observed subjective complaints or objective memory performance remains unclear. Psychosocial and environmental factors should also be taken into consideration, as pregnancy represents a major life transition characterised by substantial changes in stress levels, emotional demands, and cognitive load, all of which may influence both subjective and objective memory functioning [36].

Understanding the evidence on pregnancy-related memory functioning first requires a brief overview of the main memory systems and processes, as described in the next section, because different forms of memory are differentially affected during pregnancy. The following subsections will therefore examine memory impairment during pregnancy, considering subjective reports and objective performance, and the potential role of biological, psychosocial, and environmental factors in shaping memory functioning during this period.

### 3.1. Memory and Its Assessment

Memory is a complex cognitive function that involves the encoding, consolidation, and retrieval of information and plays a central role in shaping individual identity and everyday functioning [37].

Memory is not a unitary construct. Rather, it comprises different systems and processes [38]. Based on its duration, memory can be classified into short-term memory (STM), which refers to the temporary maintenance of information, and long-term memory (LTM), which enables information to be retained over extended periods [39]. Closely related to STM is working memory, which involves the active maintenance and manipulation of information required for ongoing cognitive tasks [40]. Based on the type of information processed, working memory can be further distinguished into verbal and visuo-spatial. Depending on the typology of stored memory, LTM can be classified into explicit (or declarative) memory, which supports the conscious recollection of facts, events, or semantic knowledge, and implicit (or procedural) memory, which supports the storage of information about skills and habits without conscious awareness, such as driving a car or riding a bike [41]. Declarative memory may involve both verbal and visuo-spatial information, as well, and includes retrospective memory, which refers to the ability to remember previously learned information or past events [42]. Memory functioning also encompasses prospective memory, which refers to the ability to remember to carry out intended actions in the future [42]. Importantly, some forms of memory, particularly working memory or prospective memory, depend more heavily on executive functions [43,44], such as monitoring, inhibition, and cognitive flexibility.

Memory can be assessed both objectively, through cognitive tasks, and subjectively, through questionnaires or self-reports of everyday memory functioning. Working memory is commonly assessed through verbal and visuo-spatial tasks, such as forward and backward digit span [45,46]. Declarative (retrospective) memory is commonly assessed through recall and recognition tasks [47]. Recall tasks require the retrieval of previously learned information without external cues and may be administered immediately after learning (immediate recall) or following a delay (delayed recall), whereas recognition tasks require the identification of previously encountered information among presented stimuli. Prospective memory is typically evaluated subjectively or through tasks requiring participants to remember and execute intended actions after a delay or in response to specific cues [48].

These distinctions are important because evidence suggests that not all memory systems are equally affected during pregnancy, with some domains appearing more vulnerable than others [5,7,29].

### 3.2. Memory Impairment During Pregnancy

#### 3.2.1. Subjective Memory Difficulties

Overall, the literature on subjective cognitive difficulties during pregnancy is based predominantly on anecdotal accounts and self-report questionnaire studies. Evidence from self-report measures consistently indicates high prevalence rates of perceived memory impairment among pregnant women [31], typically involving everyday lapses, such as forgetting intended purchases or entering a room without recalling the purpose. Brett and Baxendale [2] reported that approximately 50–80% of pregnant women experience subjective cognitive complaints, with forgetfulness and perceived memory difficulties being the most frequently reported symptoms, alongside reduced concentration and distractibility. These complaints are most often reported during the second and third trimesters of pregnancy [49].

Empirical findings support the widespread nature of these perceptions. For example, using structured interviews combined with self-rated memory scales and qualitative reports, Brindle et al. [50] found that pregnant women rated their memory functioning as worse than usual and significantly poorer than that of non-pregnant controls, with the most pronounced perceived impairments observed among primiparous women (i.e., first-time mothers) during the second trimester. Other studies have confirmed these findings, with Logan et al. [51] reporting greater self-reported memory complaints in pregnant women compared to non-pregnant controls across multiple cognitive domains, as assessed by self-report questionnaire measures.

#### 3.2.2. Objective Memory Impairment

Objective evidence supporting memory deficits during pregnancy is mixed, despite a growing body of empirical research [7]. A few studies have reported mild and domain-specific impairments [52,53], particularly during late gestation. These effects have been most consistently observed in verbal memory tasks, especially free recall [54,55,56]. For example, Keenan et al. [57] found declines in immediate and delayed paragraph recall from the second to the third trimester. Onyper et al. [53] reported poorer verbal fluency, but no differences in working memory, and these effects were no longer significant after controlling for verbal IQ (Intelligence Quotient), indicating limited clinical relevance. Some studies have also reported reduced performance on prospective memory tasks [31,58]. However, several studies have found no evidence of impairment on memory tasks despite women’s self-perceptions of decreased memory function [3,50,51,59,60,61,62].

Systematic reviews and meta-analyses provide a more integrative perspective. Henry and Rendell [5] conducted a meta-analysis of 14 studies comparing pregnant and/or post-partum women with healthy matched controls on behavioural measures of memory. They reported small impairments in pregnant women on measures of immediate free recall (r = −0.21), delayed free recall (r = −0.22), and backward digit span (r = −0.16), whereas no significant differences between pregnant and non-pregnant women were observed for STM, recognition memory, or implicit memory. Davies et al. [7] subsequently expanded the evidence base to 20 studies and considered general cognition, memory, executive functioning, and attention. They reported a small-to-moderate decline in memory performance in pregnant women compared with non-pregnant controls (overall SMD = 0.48, 95% CI 0.04–0.92), with larger differences in cross-sectional comparisons during the third trimester (SMD = 1.47, 95% CI 0.27–2.68) and smaller longitudinal declines between the first and second trimesters (SMD = 0.33, 95% CI 0.12–0.54). However, the cross-sectional estimates were characterised by substantial heterogeneity, and performance generally remained within the normal range. More recently, Younis et al. [29] qualitatively synthesised 31 studies but did not calculate pooled effect sizes because of heterogeneity in study outcomes. They confirm only a slight memory decline, with effects most evident in verbal recall and prospective memory, while other memory domains show only minor or negligible changes.

Importantly, these reviews are based on substantially overlapping primary evidence rather than independent sets of studies. Both Davies et al. [7] and Younis et al. [29] expanded upon the evidence synthesised in earlier reviews by incorporating more recently published studies, while also applying somewhat different eligibility criteria and review methods. Their apparently differing estimates therefore reflect not only the accumulation of new evidence, but also differences in eligibility criteria, cognitive domains, and synthesis methods.

#### 3.2.3. Subjective vs. Objective Memory Impairment

Overall, evidence from both subjective reports and objective performance indicates pregnancy-related memory changes [29]. Self-reported difficulties are highly prevalent, but objective assessments reveal only subtle, heterogeneous, and largely domain-specific alterations. Memory processes requiring greater effort or executive functions, such as free recall and prospective memory, are most affected, whereas STM, recognition, and working memory remain largely intact [5,7,29]. This discrepancy highlights that perceived memory lapses cannot be fully explained by objective deficits alone and underscores the importance of interpreting subjective memory complaints within a broader biopsychosocial framework.

Taken together, current evidence suggests that perceived memory difficulties during pregnancy likely arise from an interaction between subtle memory changes and contextual factors, rather than from marked neurocognitive impairment. Identifying moderating processes (such as biological and psychosocial factors, and sleep disruption) may therefore be important for understanding individual differences in both subjective experience and objective memory performance during pregnancy.

### 3.3. The Role of Biological Factors

Within the biopsychosocial framework, the biological domain refers to biological and physiological processes that contribute to an individual’s health and functioning. In the context of cognition, biological factors encompass mechanisms related to the structure and function of the nervous system, which are thought to support behaviour and cognitive processes, including memory [38,63]. More broadly, biological factors may include genetic, endocrine, neural, immune, and molecular mechanisms. However, given the scope of the present review, we focus specifically on two domains that have received considerable attention in the pregnancy literature: hormonal changes and brain plasticity. In particular, we examine the potential role of pregnancy-related hormonal fluctuations and structural and functional brain reorganisation, as documented by neuroimaging studies, in shaping memory functioning during gestation. Throughout this review, these processes will be collectively referred to as ‘biological factors’, while acknowledging that the broader biological domain encompasses additional mechanisms that are beyond the scope of the present work.

#### 3.3.1. Hormonal Fluctuations

Pregnancy induces adaptive changes in maternal physiology and brain function that support gestation and caregiving, with well-documented effects on mood, motivation, and behaviour [29,64,65]. This period is characterised by profound fluctuations in several hormones known to modulate learning and memory, including oestrogens, progesterone, cortisol, vasopressin, oxytocin, corticosteroids, and endogenous opioids [33,34,66], all of which undergo substantial changes across gestation [50,67,68]. Such hormonal alterations provide a plausible neuroendocrine basis for pregnancy-related memory changes. Given the established influence of hormones and neurotransmitters on neural plasticity and cognitive processing [69], it has been proposed that memory difficulties commonly reported during pregnancy may, at least in part, be driven by hormonal fluctuations [70]. However, empirical evidence directly linking hormonal concentrations to cognitive performance during pregnancy remains limited, inconsistent, methodologically heterogeneous and concentrated predominantly in late pregnancy.

An overview of studies directly examining associations between maternal hormone concentrations and cognitive performance is displayed in Supplementary Table S1. Early work generally provided little support for a simple linear relationship. Silber et al. [71] found temporary peripartum reductions in memory and attention but no significant association between cognitive performance and circulating oxytocin. Similarly, Buckwalter et al. [54] found that none of the measured steroid hormones consistently predicted cognitive performance during late pregnancy and the early postpartum period. Although isolated associations were observed, serum oestradiol and progesterone were not significantly related to memory performance, and circulating steroid concentrations did not reliably explain the cognitive differences identified.

One possible explanation for these null findings is that some hormone–cognition relationships may be nonlinear. Buckwalter et al. [54] proposed that steroid hormones could exert an inverted-U-shaped influence on cognition, such that the exceptionally high concentrations reached during pregnancy may not produce the cognitive benefits associated with moderate concentrations. In this context, the positive cognitive effects of oestrogens reported across the menstrual cycle or with oestrogen replacement therapy may not be generalised to the perinatal period [54]. Consistent with this possibility, Henry and Sherwin [52] found both linear and quadratic associations between hormonal concentrations and cognition. Cortisol demonstrated an inverted-U-shaped relationship with verbal recall during late pregnancy and postpartum, while prolactin was associated with paragraph recall and executive performance. These findings indicate that analyses restricted to linear correlations may overlook more complex hormonal effects.

Other longitudinal findings have also been mixed. Glynn [72] reported that trajectories of maternal cortisol and oestradiol across gestation were associated with decreases in verbal recall, although the hormones showed effects in different directions (see Table S1). In contrast, Farrar et al. [73] observed a reduction in spatial recognition memory during pregnancy, but found no significant associations between cognitive performance and plasma oestradiol, progesterone, cortisol, prolactin, dehydroepiandrosterone sulphate, or sex hormone-binding globulin.

More recent studies provide stronger, although still not conclusive, evidence concerning oestradiol. Hampson et al. [49] found that higher serum oestradiol concentrations were associated with better working memory performance during late pregnancy. Amiel Castro et al. [74] subsequently found a small positive association between free oestradiol and digit span performance in late pregnancy after adjustment for age, education, body mass index, depressive symptoms, parity, sleep, and other hormones. Progesterone and cortisol were not significant predictors in the adjusted models. Collectively, these studies suggest a possible positive association between oestradiol and working memory, but differences in cognitive tasks, hormone fractions, assay methods, sampling schedules, and covariate adjustment limit direct comparison (for more details see Table S1).

The predominance of oestradiol in this literature should not be interpreted as evidence that cortisol, progesterone, and oxytocin are unimportant. Rather, these hormones have received less focused, hypothesis-driven investigation as independent predictors of cognition. Oestradiol entered pregnancy research from an already extensive literature and remains relatively stable across the day. Cortisol, by comparison, varies according to time of day, acute stress exposure, gestational stage, and sampling conditions [75], and its associations with cognition may therefore be nonlinear. Reliable investigation may consequently require repeated or time-standardised sampling rather than a single blood measurement. Progesterone has frequently been measured alongside oestradiol but has rarely been the primary, theory-driven hormone of interest. Oxytocin presents further challenges because its secretion is pulsatile and strongly dependent on reproductive and social context, particularly around labour, birth, and lactation [76,77]. Peripheral oxytocin estimates are also sensitive to sample preparation and assay procedures [78] and may not provide a straightforward index of central oxytocin signalling. Moreover, most pregnancy–cognition studies have employed conventional episodic memory, working memory, or executive function measures, whereas oxytocin may be more relevant to social cognition, emotional salience, and caregiving-related processing [79]. These methodological and conceptual issues may partly explain the relative scarcity and inconsistency of findings for these hormones.

Taken together, current evidence suggests that while hormonal fluctuations during pregnancy provide a plausible biological basis for memory changes, direct and consistent links between specific hormones and objective memory performance have yet to be firmly established. The available evidence does not support a direct single-hormone explanation. Hormonal influences are more likely to operate through nonlinear, interacting, and context-dependent pathways involving mood, stress, sleep, and gestational timing. This complexity highlights the need for integrative approaches that consider hormonal, psychological, and social and environmental influences when examining cognitive functioning in this period.

#### 3.3.2. Structural and Functional Brain Reorganisation

Alongside hormonal adaptations, the maternal brain undergoes significant plasticity during pregnancy [36,80,81]. Evidence from animal models indicates that hormonal changes play a key role in shaping the maternal brain [82], and consistent findings in humans show that hormonal fluctuations are associated with structural and functional brain alterations [83]. Importantly, evidence from other reproductive hormonal states indicates that endogenous hormonal fluctuations are associated with changes in intrinsic functional connectivity and network reorganisation, particularly within large-scale networks involved in higher-order cognitive functions [84]. Emerging neuroimaging research has begun to clarify these changes during gestation [83]. Exponential increases in oestrogen and progesterone may influence cortical and limbic network activity, potentially affecting cognitive and affective processing [29,85,86].

Structural Magnetic Resonance Imaging (sMRI) provides high-spatial-resolution information on anatomical brain changes. Research suggests that pregnancy is characterised by widespread brain remodelling, including reductions in overall brain volume, grey matter, and cortical thickness, alongside increases in white matter microstructural integrity, ventricular volume, and cerebrospinal fluid. Anatomical alterations have also been reported in regions involved in executive functioning, social cognition, emotion regulation, and memory, including the hippocampus and amygdala [81,87,88,89].

Alongside structural imaging approaches, functional neuroimaging techniques provide complementary information on brain function. In particular, resting-state and task-based functional Magnetic Resonance Imaging (fMRI) allow the investigation of large-scale functional brain networks and their connectivity. fMRI studies conducted during pregnancy suggest alterations in brain systems supporting reward and motivation, salience and fear, social cognition and emotional processing [87,90,91]. These networks are consistently engaged in response to infant-related stimuli and are thought to support caregiving behaviours, including sensitivity to emotional cues and motivational responses to the infant [87]. Rather than reflecting a general cognitive decline, these changes are increasingly interpreted as part of a selective and adaptive reorganisation of the maternal brain, particularly within networks supporting social cognition and emotional processing that are relevant for caregiving behaviours [36,81,89,92,93]. Consistent with this interpretation, some studies report enhanced social and emotional processing during pregnancy, including improved recognition of faces and emotional cues [94,95].

While current evidence on functional brain reorganisation during pregnancy has been obtained primarily using fMRI, other functional neuroimaging techniques may provide complementary insights into pregnancy-related brain adaptations. Magnetoencephalography (MEG), for example, which directly measures neural activity with high temporal resolution, could allow the investigation of oscillatory activity and rapid changes in brain dynamics. Although MEG has not yet been widely applied during pregnancy, studies conducted across the menstrual cycle have shown that hormonal fluctuations are associated with changes in resting-state neural activity [96], as well as brain flexibility and connectome stability [97]. These findings highlight the potential of MEG as a complementary tool for investigating functional brain adaptations in future pregnancy research.

The precise mechanisms underlying these biological changes remain unclear. Notably, evidence from other developmental periods, such as adolescence, indicates that similar patterns of brain reorganisation may reflect hormonally driven processes of neural refinement, including synaptic pruning and network specialisation [81,92,98,99]. Consistent with this, pregnancy-related brain changes appear to be specific to the biological processes of gestation, as they are not observed in fathers [81], highlighting the potential role of hormonal factors in driving these adaptations.

The functional implications of this remodelling have yet to be fully understood. While some evidence links pregnancy to declines in memory performance, other work highlights pregnancy and early motherhood as periods of heightened neuroplasticity that may support adaptive cognitive changes [100]. Overall, although evidence remains limited, current neuroimaging research suggests that dynamic brain reorganisation may contribute to the subtle and domain-specific memory changes observed during pregnancy.

Importantly, this evidence alone does not provide a comprehensive explanation for the cognitive changes observed during pregnancy, suggesting that additional factors should be considered. In this context, these changes may be better understood as embedded within a broader interplay of psychosocial and environmental influences.

### 3.4. The Role of Psychosocial and Environmental Factors

Biological changes during pregnancy occur alongside substantial psychosocial and environmental adjustments that may also contribute to cognitive changes [36]. Within a biopsychosocial framework, these factors refer to interrelated psychological, social, and contextual influences that may shape subjective perception of memory functioning during pregnancy. Psychological factors include emotional states and potential psychopathology, personality traits, concerns related to pregnancy and the transition to motherhood, and individual differences in the psychological adaptation to these experiences. Social factors encompass interpersonal relationships, including partner and family dynamics, perceived social support, social roles, and societal expectations surrounding motherhood and maternal memory functioning. Finally, environmental and contextual factors refer to broader life circumstances, such as work demands, financial strain, and housing, which may become particularly salient during the transition to parenthood.

Although the transition to parenthood is often experienced as a positive and meaningful life event, it can also evoke fears, concerns, anxiety, and stress, with significant implications for maternal emotional and psychological well-being [101]. In this context, subjective memory complaints may be amplified by psychological factors such as low mood, anxiety, and personality traits, such as neuroticism [102]. Moreover, the transition to motherhood involves notable emotional adaptation, including the adoption of new identities and social roles [36], as well as increased concerns related to the child’s health and future parenting responsibilities [103]. Pregnant women may also experience additional stressors, such as intimate relationship changes, body image concerns, and worries about childbirth [104,105]. However, the nature and intensity of these experiences may vary according to individual differences, including, for example, parity. First-time mothers often report greater uncertainty regarding the transition to motherhood, higher levels of childbirth-related fear and anxiety, lower childbirth self-efficacy [106], and greater concerns about bodily changes and maternal role attainment [107] compared to multiparous women (i.e., women who already have children). Differences also emerge in perceived social support. Primiparous women tend to report lower perceived levels of family and social support, whereas multiparous women may express greater concerns about a lack of social support [107]. Indeed, multiparous women must often balance pregnancy with the care of older children and other family responsibilities [108], potentially increasing daily cognitive and emotional load.

Beyond these factors, emerging evidence suggests that pregnancy may also involve shifts in motivational and attentional priorities. Specifically, some women appear to allocate greater attentional resources to infant-related information, potentially reducing engagement with laboratory cognitive tasks [31], while enhancing memory for personally relevant stimuli [109]. These changes may reflect adaptive reallocation of attentional resources under increased psychosocial demand. However, these findings are not consistently observed and may reflect individual differences rather than a general pattern.

Subjective memory complaints may also be amplified by societal expectations surrounding pregnancy-related memory decline [6]. Continuous re-evaluation and heightened awareness of memory ability may also be amplified by the societal expectation of maternal cognitive decline in some cultures [4,6], contributing to confirmation bias [36].

Furthermore, the arrival of a newborn in a family often entails practical and economic adjustments, including changes in work demands, occupational activity, and financial resources [110]. Concerns about income stability, employment, and housing [103] may arise at a time when family expenses are increasing, potentially adding to cognitive load [110]. These challenges may be particularly relevant for multiparous women, who may experience additional caregiving and family-management demands during pregnancy [108], which could contribute to greater everyday cognitive demands. Despite the plausibility of these influences, the role of work-related and economic stressors in shaping cognitive functioning during pregnancy remains largely unexplored.

Psychosocial and environmental factors associated with memory complaints during pregnancy have received considerably less attention than biological mechanisms. Although research in this area remains limited, emerging evidence suggests that societal expectations and contextual stressors may influence self-perceived memory functioning [111] and interact with biological processes in shaping, at least, subjective memory outcomes.

Together, these findings suggest that subjective and objective memory difficulties during pregnancy emerge from a complex interplay between biological processes and broader psychosocial and environmental demands. Within this multifactorial framework, sleep may represent a particularly relevant, but often underexplored, factor, given its fundamental role in memory functioning [12] and the high prevalence of poor sleep health during pregnancy [18].

## 4. Sleep Health During Pregnancy and Its Relationship with Memory

Sleep and wake patterns are significantly challenged during the perinatal period. New mothers are exposed to possible chronic sleep disruption and fragmentation during pregnancy, acute sleep deprivation during labour, and the immediate postpartum period [112]. Up to 78% of women complain about disturbed sleep during the third trimester of pregnancy [18]. However, pregnant women experience alterations in sleep duration, quality, and pattern across all gestational weeks [113]. Common issues during all three trimesters include short sleep duration, poor sleep quality, and insomnia symptoms, which persist and worsen during the postpartum period [113]. Multiple factors contribute to these difficulties, such as anatomical and hormonal changes [114,115], physical discomfort [19], changes in social rhythms (e.g., early interruption of work activities) [116], and heightened psychological arousal (e.g., increased worries or excitement) [117]. Women cite several reasons for sleep disturbance during pregnancy, including increased need to urinate, difficulty finding comfortable sleeping positions, and body aches [118]. Alongside these concerns, mood disturbances [119], older age [120], income [121], trimester or gestational age [122,123], ethnicity, and parity [124,125] have been reported to influence sleep quality during pregnancy [126].

Therefore, the peripartum period is characterised by profound biopsychosocial changes that may challenge the optimal functioning of all dimensions of sleep health. In the following subsections, each sleep health dimension will be examined with a specific focus on pregnancy [116] and in relation to memory performance. For a broader overview of cognitive and emotional correlates of poor sleep during pregnancy, see Baldi et al. [127].

### 4.1. Sleep Health Dimensions and Memory Impairment During Pregnancy

#### 4.1.1. Sleep Satisfaction

Sleep satisfaction refers to the subjective evaluation of one’s sleep quality, that is, the perception of having slept in a refreshing and restorative way. Several studies investigated the subjective perception of pregnant women’s sleep quality, which is not the same construct as satisfaction. A meta-analysis shows that pregnant women experience poorer sleep quality compared to the general population, which tends to worsen from the second to the third trimester [123].

To the best of our knowledge, no studies have specifically examined the relationship between sleep satisfaction and memory in pregnant women. However, several studies have addressed the related construct of self-reported sleep quality. Berndt and colleagues [128] reported significant positive correlations between self-reported sleep difficulties—measured with the Pittsburgh Sleep Quality Index (PSQI) [129]—and the percentage of false alarms, an index of false memory formation, assessed with the Deese–Roediger–McDermott (DRM) paradigm [130,131]. The same study also found a significant negative association with verbal working memory performance, assessed using the WAIS Digit Span task [132]. In line with these findings, Zhang et al. [133] observed that pregnant women with self-reported poor sleep quality (PSQI) reported more subjective memory impairment—assessed with the Prospective and Retrospective Memory Questionnaire (PRMQ) [134]—compared to women reporting good sleep quality. Overall, these findings may suggest that a subjective perception of poor sleep quality is associated with both poorer performance on memory tasks and a more negative subjective evaluation of memory functioning.

#### 4.1.2. Daytime Alertness

Daytime alertness refers to the ability to maintain attentive wakefulness during the day. This dimension is commonly assessed in its negative aspect, i.e., sleepiness—difficulty in maintaining an adequate level of functioning during the day. The first pregnancy-related change in sleep that many women notice is an increase in daytime sleepiness, often accompanied by a subjective sense of physical and mental fatigue [135]. Although pregnant women often complain of feeling tired, this term can refer to two distinct concepts: sleepiness, which is closely linked to the urge to sleep, and fatigue, which can be defined as a sensation of weariness, weakness, and energy depletion [136]. Distinguishing between fatigue and daytime sleepiness can be complex, but it has important clinical and diagnostic implications, as these phenomena have different causes and consequences [137]. A third of pregnant women report significant daytime sleepiness [19]. Furthermore, pregnant women are more likely to report excessive daytime sleepiness compared to non-pregnant controls [138].

Evidence on daytime alertness and memory during pregnancy is scarce. One study reported that pregnant women experiencing daily sleep-related disturbances, assessed using the PSQI, showed greater subjective memory impairment on the PRMQ compared to women without such disturbances [133], highlighting a potential link between daytime sleepiness and perceived cognitive difficulties.

#### 4.1.3. Sleep Timing and Regularity

Sleep timing refers to the positioning of the major sleep episode within the 24 h day, including bedtime and wake time, which define the window in which sleep need should be satisfied [22]. Instead, sleep regularity refers to the consistency of sleep–wake patterns across weekdays and weekends, and the degree to which sleep timing varies between them. There are inter-individual differences in optimal sleep timing, known as circadian typology [139]. Although little is known about circadian timing during pregnancy, emerging evidence indicates that circadian rhythms undergo notable changes across this period [116]. Specifically, women appear to shift sleep onset earlier during the first and second trimesters compared to the pre-pregnancy period, whereas circadian timing tends to return to its pre-pregnancy pattern during the third trimester [21,140].

To the best of our knowledge, no studies have directly examined the relationship between sleep timing or regularity and memory performance in pregnant women, representing a notable gap in the literature.

#### 4.1.4. Sleep Efficiency

Sleep efficiency represents the ratio between time spent sleeping and time spent in bed. A sleep efficiency index of 85% is generally considered indicative of good sleep efficiency [27]. This dimension also includes sleep onset latency, wake after sleep onset or nighttime awakenings, and early morning awakenings, which constitute the most common complaints among insomnia patients. Insomnia symptoms are common during pregnancy, including difficulties in falling or staying asleep and early awakenings, which directly impact overall sleep efficiency [116]. The risk of insomnia is estimated to be higher later in pregnancy compared to earlier [141], and 14–27% of expectant mothers report clinically significant insomnia symptoms [19,142]. A study involving 325 pregnant women reported increased nighttime awakenings and reduced sleep efficiency from the first trimester onward, continuing throughout pregnancy [120].

Sleep efficiency has received comparatively more attention, although findings are largely non-significant. Hampson et al. [49] found no significant associations between nighttime awakenings and visuo-spatial working memory, assessed using the Spatial Working Memory [143] and Self-Ordered Pointing [144] tasks. Similarly, the study by Zhang and colleagues [133] found no significant differences in subjective memory impairment (PRMQ) between women reporting poor sleep efficiency (PSQI) and those with good sleep quality. Another study using polysomnography [145] found a non-significant correlation between wake after sleep onset and procedural memory (Finger-tapping task) [146] and visuo-spatial declarative memory (Object-location memory task) [147], or false memory formation (DRM paradigm).

#### 4.1.5. Sleep Duration

Sleep duration refers to the quantity of sleep in 24 hours. It has been reported that approximately 28% of pregnant women sleep less than 7 hours per night [148], which is below the recommended sleep duration for the adult population [149]. A study involving 325 pregnant women found that perceived total sleep time increased during the first trimester, and then slightly decreased during the second trimester, followed by a substantial decrease during late pregnancy [120]. Objective measurements of sleep using polysomnography indicate reduced sleep duration, consistent with subjective reports [150].

Studies examining sleep duration have likewise reported predominantly null findings. No significant associations were observed between sleep duration and visuo-spatial working memory in the study by Hampson et al. [49], nor between actigraphy-measured sleep duration and verbal declarative memory assessed with the Rey Auditory Verbal Learning Test [151] in a study by Nuckols et al. [152]. Consistent with these results, Zinke and colleagues [145] found non-significant correlations between polysomnography-measured sleep duration and procedural memory (Finger-tapping task), visuo-spatial declarative memory (Object-location memory task) or false memory formation (DRM paradigm).

#### 4.1.6. Pre-Sleep Behaviours

Pre-sleep behaviours refer to activities performed in the hour before going to bed. They may promote or inhibit sleep and regularity of bedtime routines, such as a consistent sleep schedule and a bedtime routine, or the use of technology in the hour before going to bed [116]. According to the International Classification of Sleep Disorders [153], the main categories of nocturnal lifestyle habits that can result in poor sleep include an inconsistent sleep–wake schedule, an unconducive sleeping environment, the use of stimulants such as coffee or nicotine, and poor pre-sleep behaviours such as studying or working in bed. However, there is a lack of understanding of how they can affect sleep quality in expectant mothers. A study on a cohort of pregnant women assessed various nocturnal behaviours and their associations with sleep quality, finding that meal intake after 8 p.m. and artificial light exposure at night were associated with poor sleep. On the contrary, physical activity and screen viewing before bedtime had no significant association with sleep quality [154].

To the best of our knowledge, no studies have explored the association between pre-sleep behaviours and memory performance during pregnancy. In general, more research on pre-sleep behaviours in pregnancy is needed.

Table 1 visually summarises the available evidence on the associations between each sleep health dimension and subjective and objective memory functioning in pregnant women, highlighting the consistency and statistical significance of findings for each dimension of sleep health. A more detailed study-by-study summary, including study design, sample characteristics, sleep and memory measures, main findings, and study limitations, is provided in Supplementary Table S2.

## 5. Discussion

This narrative review aimed to provide an overview of memory difficulties during pregnancy, with particular attention to the potential biological and psychosocial mechanisms underlying these alterations. It also examined sleep health as a specific and potentially modifiable factor within this framework by synthesising the existing literature on sleep health, memory, and their interactions during gestation. Overall, the literature suggests that pregnancy is associated with widespread subjective memory complaints, whereas objective memory changes are generally subtle, domain-specific, and often within normative ranges [5,7,29]. Biological evidence indicates that pregnancy is characterised by substantial hormonal fluctuations and brain reorganisation, both of which provide a plausible biological context for memory changes [36,70]. However, current findings do not support consistent associations between biological factors and memory decline [49,54]; rather, some studies point to potential adaptive changes, suggesting that pregnancy may involve forms of neuroplasticity that support cognitive functioning in certain contexts [100].

The mechanisms underlying these biological changes remain only partially understood, with evidence suggesting hormonally driven processes of neural reorganisation that appear to be specific to pregnancy. At the same time, societal perspectives suggest that expectations and stereotypes surrounding “baby brain” may influence women’s perceptions and reports of memory difficulties [4,6]. Taken together, these findings indicate that the discrepancy between subjective complaints and objective performance is unlikely to be explained by biological mechanisms alone but rather reflects the interaction between subtle biological changes and psychosocial and contextual factors. This underscores the importance of interpreting subjective memory changes during pregnancy within a broader biopsychosocial framework, rather than as evidence of a generalised cognitive decline.

Methodological considerations may also contribute to this discrepancy. Although objective measures provide reliable assessments of specific memory domains, their ecological validity may be limited, as they are administered under highly controlled conditions that differ substantially from the cognitive demands encountered in everyday life [155]. Consequently, subjective memory complaints may reflect real-world cognitive challenges that are only partially detected by conventional laboratory-based assessments.

In parallel, sleep health appears to be consistently compromised during pregnancy, with evidence indicating deterioration across multiple dimensions, especially self-reported sleep quality, duration, efficiency, and daytime alertness. Despite the well-established role of sleep in memory processes in the general population [10,12,13,14], the evidence linking sleep to memory functioning during pregnancy remains limited and heterogeneous. The available studies report associations rather than causal relationships, with some suggesting that poorer self-reported sleep quality and increased daytime sleepiness are related to worse memory performance [128] or increased subjective memory complaints [133], while others report null findings for sleep duration and efficiency [49,145,152]. Although only a few studies have directly examined the relationship between dimensions of sleep health and memory during pregnancy, the evidence presented in this review indicates that pregnancy is characterised by dynamic brain reorganisation, while broader neuroimaging research in non-pregnant populations has shown that sleep disruption affects cognitive [156,157] and emotional [158] functioning, as well as resting-state [159] and functional brain connectivity [160]. For instance, recent MEG findings suggest that sleep deprivation may alter functional connectome stability [161]. Together, these findings raise the possibility that sleep health may influence pregnancy-related memory functioning through biological mechanisms that remain largely unexplored. Overall, these findings point to sleep as a relevant, yet underexplored, pathway through which pregnancy-related changes could influence memory functioning. However, current evidence does not allow firm conclusions regarding the directionality or mechanisms underlying this relationship.

An additional perspective that may help explain the heterogeneity of findings concerns the role of executive functions. Interestingly, some of the observed inconsistencies in the relationship between sleep and memory during pregnancy may be better understood by considering the role of executive functions, including cognitive inhibition, monitoring and flexibility, as well as working memory, a process that is variably conceptualised as part of both memory systems and executive functioning [162]. Memory performance, particularly in complex or effortful tasks, relies heavily on executive functions [43,44]. The reviewed literature suggests that the subtle cognitive changes reported during pregnancy are more likely to emerge on tasks with greater executive demands [5,7,29]. In line with this, neuroimaging findings suggest alterations in brain systems supporting executive functioning during pregnancy, providing a potential biological basis for these observations [87]. Moreover, recent evidence suggests that longer sleep duration is associated with better cognitive inhibition, even when no direct association with memory outcomes is observed [152]. Together, these findings raise the possibility that executive functions may contribute to the relationship between sleep health and memory functioning during pregnancy. However, the available evidence is still limited and not sufficient to support definitive conclusions regarding a role of executive functions in this relationship.

### 5.1. Gaps in the Available Literature and Limitations of the Present Review

Several limitations emerge from the existing body of research. First, most studies provide only cross-sectional data, limiting the ability to infer temporal or causal relationships between sleep and memory. There is a lack of longitudinal studies examining how memory changes evolve across pregnancy and the postpartum period in relation to sleep health. Consequently, the extent to which sleep health represents a causal, maintaining, or merely associated factor in gestational memory changes remains to be clarified. Moreover, the available evidence is based on a limited number of studies, many of which included relatively small samples, potentially reducing statistical power to detect meaningful associations. Therefore, the predominance of non-significant findings for some dimensions of sleep health should not be interpreted as evidence of the absence of an association, but rather as reflecting the current limitations of the available evidence. Publication bias cannot be excluded, given the limited number of published studies in this area. Second, studies differed considerably in the assessment of both sleep and memory. In particular, sleep was assessed using subjective questionnaires, as well as objective methods, such as actigraphy and polysomnography, which capture different aspects of sleep and are not directly interchangeable. This methodological heterogeneity complicates direct comparisons across studies and may partly contribute to inconsistent findings. Third, several dimensions of sleep health, such as sleep timing, regularity, and pre-sleep behaviours, remain largely unexplored or underexplored in relation to memory functioning during pregnancy, representing important gaps in the literature. Fourth, more integrative approaches combining sleep, memory performance, biological measures, and psychosocial variables within the same study are scarce. Given the complex interplay between biological and psychosocial factors, examining these processes in isolation may neglect important mechanisms underlying memory changes. Finally, research has predominantly focused on maternal populations, with limited attention to the broader family context. This narrow focus may overlook relevant psychosocial dynamics that could influence both sleep and memory functioning.

In addition to the limitations of the available evidence, some considerations regarding the present review should be acknowledged. Although a structured literature search was conducted for studies examining the association between sleep and memory, and additional targeted searches were undertaken to support the broader biopsychosocial framework, this review was conducted using a narrative approach. This methodology enabled the integration of evidence from diverse research domains and the development of a comprehensive conceptual framework, but it does not provide the level of methodological standardisation or reproducibility of a systematic review. Consequently, the possibility of selection bias in the inclusion and interpretation of the evidence cannot be entirely excluded, and no formal quality or risk of bias assessment of the included studies was performed. Furthermore, the conclusions of this review should be interpreted in light of the potential publication bias, as studies reporting statistically significant or positive findings are generally more likely to be published and, therefore, represented in the available evidence.

### 5.2. Future Directions and Clinical Implications

Limitations of the available literature also point to several important directions for future research and clinical practice. Future studies should adopt longitudinal designs with trimester-specific assessments and postpartum follow-ups to better capture trajectories of change in both sleep and memory across pregnancy and the postpartum period, thereby helping to clarify potential causal relationships between these processes. Furthermore, integrating objective and subjective measures of both sleep and memory would allow a more comprehensive understanding of these processes. Sleep should be assessed using both subjective methods, such as validated questionnaires (e.g., PSQI) or sleep diaries, and objective measures, including actigraphy or polysomnography. Combining these complementary assessment methods within the same study may also facilitate the interpretation of discrepancies between subjective and objective sleep measures and improve the comparability of findings across studies. Similarly, memory functioning should be evaluated using both self-report measures of everyday memory functioning and standardised neuropsychological assessments to better characterise the discrepancy between perceived and objectively measured cognitive changes.

Additionally, more research is needed to examine sleep health using a multidimensional framework, moving beyond the predominant focus on sleep disturbances and insomnia. Investigating specific sleep health dimensions, particularly those that remain understudied, may provide a more comprehensive understanding of how different aspects of sleep contribute to memory functioning during pregnancy and help identify targets for intervention [116].

Given the multifactorial nature of pregnancy-related changes, future research would benefit from multimodal approaches that simultaneously consider hormonal fluctuations, brain plasticity, psychosocial stressors, and sleep health. Beyond adopting multidimensional assessments of sleep health and combining subjective and objective measures of memory, such studies should characterise biological processes through repeated assessments of hormonal markers (e.g., oestradiol, progesterone, cortisol). Psychosocial influences could be evaluated using validated measures of mood, anxiety, perceived stress, emotion regulation, and other relevant psychological factors. Social and contextual influences, including perceived social support, occupational and socioeconomic conditions, and women’s experiences of pregnancy and adaptation to the maternal role, should also be considered using both quantitative and qualitative approaches. Finally, objective measures of brain function, including neuroimaging techniques such as fMRI and MEG, should be integrated to investigate the neural mechanisms linking sleep health to memory functioning and to clarify whether pregnancy-related memory complaints reflect dynamic changes in functional brain organisation rather than stable memory impairment. Although it may not be feasible to include all these components within a single study, integrating multiple levels of assessment across complementary research designs would substantially improve our understanding of the mechanisms underlying pregnancy-related memory changes and help explain the considerable inter-individual variability observed in both sleep and memory outcomes during pregnancy.

Within this multimodal framework, future studies should investigate whether executive functions represent a potential mechanism linking sleep health to memory functioning during pregnancy. Such studies should simultaneously assess multidimensional sleep health, executive functions, and subjective and objective memory outcomes, while considering relevant biological, psychological, and contextual factors that may influence these relationships. In addition, research should extend beyond a mother-centric perspective and consider the role of fathers and other caregivers. The transition to parenthood involves shared responsibilities, changes in routines, and increased cognitive load that may affect sleep and cognitive functioning in both parents [163]. Including fathers in research may provide a more ecologically valid understanding of how family-level dynamics influence sleep and cognition in both parents and may help disentangle biological from contextual effects.

Finally, intervention and randomised controlled studies targeting sleep are needed to determine whether improving sleep health leads to improvements in memory functioning during pregnancy. Such studies should be adequately powered and carefully designed to account for the considerable inter-individual variability in both sleep and cognitive functioning during pregnancy. If confirmed, this would have important implications for perinatal care, including routine screening for sleep disturbances and the development of preventive interventions.

From a clinical perspective, the available findings highlight sleep health as a potentially relevant—yet still underexplored—factor in understanding memory functioning during pregnancy. Although the current evidence does not allow firm conclusions about the relationship between sleep and memory in this population, the well-established role of sleep in cognitive processes and memory functioning [12,14] suggests that sleep disturbances may plausibly contribute to the subjective memory difficulties frequently reported by pregnant women, alongside other biological and psychosocial factors discussed in this review. In this context, routine screening for sleep disturbances during pregnancy may help identify women experiencing memory complaints, even if the underlying mechanisms remain unclear. Given the modifiable nature of sleep, addressing sleep health could represent a promising avenue for supporting memory functioning. Among the different dimensions of sleep health, pre-sleep behaviours deserve particular attention because they represent potentially modifiable lifestyle factors. Although direct evidence linking pre-sleep behaviours to memory functioning during pregnancy is still lacking, promoting healthy bedtime routines may represent a low-risk strategy to improve sleep health and could be incorporated into routine antenatal care. Future studies should determine whether targeting pre-sleep behaviours may also contribute to improving maternal cognitive functioning. Overall, further research is needed to clarify the extent and nature of sleep health dimensions’ impact on memory during pregnancy before specific clinical recommendations can be established.

To facilitate interpretation, key gaps in the current literature, recommendations for future research and their expected contribution are summarised in Table 2.

## 6. Conclusions

This review highlights the complexity of memory difficulties during pregnancy, which cannot be reduced to a simple pattern of cognitive decline. Rather than supporting a generalised cognitive decline, the available empirical evidence points to a more nuanced picture in which subtle biological processes interact with psychosocial and environmental factors, while persistent assumptions about pregnancy-related memory decline may also shape scientific interpretation and public perception. Within this framework, sleep health emerges as a promising, yet still insufficiently understood, factor that may contribute to memory difficulties during this period. Importantly, these findings call for moving beyond the simplified and often stereotypical notion of “baby brain” by recognising the multifaceted nature of memory changes in expectant mothers. Clarifying the role of sleep in shaping memory outcomes represents an important step towards advancing both theoretical models and clinical practice. Future research should therefore adopt integrative and longitudinal approaches to better capture these complex interactions. In parallel, investigating modifiable dimensions of sleep health, particularly pre-sleep behaviours, may help identify practical and preventive strategies that can be translated into routine perinatal care to support maternal well-being and memory functioning.

## Figures and Tables

**Table 1:** Overall evidence on the associations between sleep health dimensions and subjective and objective memory impairment among pregnant women across available studies.

Sleep Health Dimensions	Associations Between Sleep and Subjective Memory	Associations Between Sleep and Objective Memory	Overall Evidence
Sleep satisfaction or self-reported sleep quality	Women with self-reported poor sleep quality (PSQI) report greater prospective and retrospective memory impairment (PRMQ) than those with good sleep quality [133].	Poor self-reported sleep quality (PSQI) associated with higher percentage of false memories (DRM), and poorer verbal working memory performance (digit span—WAIS) [128].	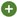 Limited significant evidence (2 studies)
Daytime alertness or sleepiness	Women experiencing daytime sleepiness (PSQI) show greater prospective and retrospective memory impairment (PRMQ) compared to women without such disturbances [133].	–	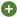 Limited significant evidence (1 study)
Sleep timing and regularity	–	–	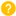 Not yet investigated
Sleep efficiency	Non-significant differences in prospective and retrospective memory impairment (PRMQ) between women reporting poor sleep efficiency (PSQI) and those with good sleep quality [133].	Non-significant associations between nighttime awakenings (one question) and visuo-spatial working memory (SPWM and SOP) [49]. Non-significant correlations between WASO (PSG) and procedural memory (finger-tapping task), visuo-spatial declarative memory (object-location memory task) or false memory formation (DRM) [145].	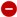 Limited non-significant evidence (3 studies)
Sleep duration	–	Non-significant associations between sleep duration (one question) and visuo-spatial working memory (SPWM and SOP) [49]. Non-significant associations between sleep duration (actigraphy) and verbal declarative memory (RAVLT) [152]. Non-significant associations between sleep duration (PSG) and procedural memory (finger-tapping task), visuo-spatial declarative memory (object-location memory task), and false memory formation (DRM) [145].	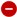 Limited non-significant evidence (3 studies)
Pre-sleep behaviours	–	–	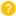 Not yet investigated

Note. DRM = Deese–Roediger–McDermott; PRMQ = Prospective and Retrospective Memory Questionnaire; PSG = polysomnography; PSQI = Pittsburgh Sleep Quality Index; RAVLT = Rey Auditory Verbal Learning Task; SOP = Self-Ordered Pointing; SPWM = Spatial Working Memory; WAIS = Wechsler Adult Intelligence Scale; WASO = Wake After Sleep Onset. The symbols (+/−/?) summarise the significance of the reported associations between sleep and memory across the included studies (+ = significant association; − = non-significant association; ? = not yet investigated).

**Table 2:** Summary of key gaps, future research directions and their expected contribution in the study of sleep health and memory functioning during pregnancy.

Current Gaps	Future Directions	Expected Contribution
Predominance of cross-sectional data	Conduct longitudinal studies across pregnancy and the postpartum period	Clarifies trajectories and potential causal relationships
Limited integration between subjective and objective measures	Combine self-report and objective assessments for both sleep and memory	Clarifies the relationship between perceived and objective memory changes in relation to both subjective and objective sleep
Predominant focus on sleep disturbances and insomnia	Adopt a positive multidimensional sleep health framework	Identifies relevant sleep dimensions and informs targeted interventions
Underexplored sleep health dimensions	Examine individual sleep health dimensions	Clarifies effects of specific sleep health dimension on memory and identifies targets for intervention
Limited integration of biological and psychosocial factors in the literature on sleep and memory	Integrate sleep, executive functions, biological, and psychosocial factors in the study of memory during pregnancy	Provides a more comprehensive understanding of the mechanisms underlying memory changes
Limited consideration of inter-individual variability and individual differences	Investigate biological, psychosocial and contextual moderators	Explains variability in sleep and memory outcomes and clarifies mechanisms underlying memory functioning during pregnancy
Predominance of mother-centric research	Include fathers and other caregivers in perinatal research	Improves understanding of family-level dynamics and helps disentangle biological and contextual influences
Lack of intervention studies examining whether improving sleep health leads to improvement in memory functioning	Conduct intervention studies and randomised controlled trials targeting sleep during pregnancy	Establishes causal links and informs clinical interventions

## Data Availability

No new data were created or analyzed in this study. Data sharing is not applicable to this article.

## Supplementary Materials

The following supporting information can be downloaded at: https://www.mdpi.com/article/10.3390/brainsci16080821/s1, Table S1: Studies examining maternal hormone concentrations and cognition during pregnancy. Table S2: Descriptive characteristics and main findings of the studies examining sleep health dimensions in relation to memory in pregnant women.
