# Supplementary material for: Beyond ‘Baby Brain’: Memory and Sleep Health During Pregnancy—A Biopsychosocial Perspective
Source: Brain Sci. 2026 Jul 31;16(8):821. doi: 10.3390/brainsci16080821 (PMC13510741; doi:10.3390/brainsci16080821)
Supplement: Supplementary file 1 [file brainsci-16-00821-s001.zip › brainsci-4446718-supplementary.pdf]

Table S1. Studies examining maternal hormone concentrations and cognition during pregnancy.

References	Study design	Sample size and assessment timing	Hormones assessed	Cognitive domains and their assessment	Main findings	Covariates and methodological considerations
**Amiel Castro et al. (2021)**	**Cross-sectional** study	**216 pregnant women** assessed at 35th-39th gestational weeks; sensitivity analysis in 155 women without clinically relevant depressive symptoms	Serum **oestradiol**, **progesterone**, **testosterone**, serum and salivary **cortisol**	**Working memory** (WAIS-III Digit Span – forward, backward, and total scores)	**Higher free oestradiol** was associated with **better total Digit Span performance** (B = 0.01, 95% CI 0.00-0.03). Progesterone and cortisol were not significantly associated. **Lower total testosterone** was related to **better backward span** in the non-depressed subgroup (B = -0.03).	Adjusted for age, body mass index, education, depressive symptoms, parity, sleep, and intercorrelations among hormones.
**Buckwalter et al. (1999)**	**Longitudinal** study	**19 pregnant women** assessed during the final two months of pregnancy and again during the early postpartum period	Serum **oestradiol**, **progesterone**, **testosterone**, **cortisol**, and **dehydroepiandrosterone**	**Episodic** (CVLT) and **semantic** (Boston Naming Test) **verbal memory**<br>**Attention** (WAIS Digit Span: forward and backward)<br>**Auditory comprehension** (modified Token Test)<br>**Executive function** (Trail-Making Tests A and B; Stroop Colour and Word Test)<br>**Visuo-perceptual performance** (JLO)	**Isolated moderate-to-large correlations** were reported **(|r|=0.48.–0.65)**, but associations were inconsistent across hormones, cognitive measures, and assessment periods and did not survive multiple-comparison correction.	Mood was assessed; small sample; multiple comparisons.
**Farrar et al. (2014)**	**Longitudinal** and **case-control** study	**23 pregnant women** (assessed once per trimester and at three months postpartum) vs 24 non-pregnant controls	Plasma **oestradiol, progesterone**, **cortisol**, **prolactin**, **dehydroepiandrosterone** sulphate, and sex hormone-binding **globulin**	**Working memory**<br>**Executive function**<br>**Spatial recognition memory** (all assessed with CANTAB)	No significant hormone-cognition associations. Spatial recognition memory was approximately 11.7% lower after the first trimester in the pregnant group, with a significant group-by-time interaction (p = 0.005).	Depressive symptoms, premorbid ability, and parity considered; small sample.
**Glynn (2010)**	**Longitudinal cohort** study	**254 pregnant women** assessed repeatedly across pregnancy vs 48 non-pregnant controls; postpartum follow-up	Plasma **cortisol**, and serum **oestradiol**	Verbal **recall** (word-pair task)<br>**Recognition** memory (face recognition paradigm)<br>**Working memory** (backward Digit Span task)	Prenatal cortisol and oestradiol predicted verbal recall in opposing directions. **Higher cortisol** was associated with **better recall** at 36 weeks (β=0.31, p<0.01) and 3 months postpartum (β=0.23, p<0.01), whereas **higher early-pregnancy oestradiol** was associated with **poorer recall** at 36 weeks (β=-0.18, p<0.01) and 3 months postpartum (-0.17, p<0.05). Together, cortisol and oestradiol explained 11% of late-pregnancy verbal-recall variance.	Trajectory-based analysis; recognition and working memory were comparatively unaffected.

References	Study design	Sample size and assessment timing	Hormones assessed	Cognitive domains and their assessment	Main findings	Covariates and methodological considerations
**Hampson et al. (2015)**	**Longitudinal** and **case-control** study	**28 pregnant women** at 34th-38th gestational weeks vs 26 non-pregnant controls; reassessed approximately 14 weeks postpartum	Serum **oestradiol**, and **cortisol**	**Visuo-spatial working memory** (SPWM and SOP tasks)<br>**Verbal memory** (paragraph recall, WAIS-III)	**Higher serum oestradiol** was moderately associated with **fewer working-memory errors** during late pregnancy (partial r=-0.45, p<0.05; adjusted β=-0.37 to -0.44). Oestradiol and depressive symptoms jointly explained 20%–27% of working-memory variance. Cortisol was not significantly associated with working memory, and no hormone effects were reported for explicit memory.	Depression severity and sleep considered; modest sample; interaction between mood and pregnancy status.
**Henry & Sherwin (2012)**	**Longitudinal** study	**55 pregnant women** vs 21 non-pregnant controls; 34th-38th gestational weeks and approximately 12 weeks postpartum	Serum **oestradiol, progesterone**, **testosterone**, **cortisol**, **prolactin**, and salivary cortisol	**Working memory** (WAIS-III)<br>**Verbal memory** (WAIS-III)<br>**Processing speed** (WAIS-III)<br>**Attention** (WAIS-III)<br>**Executive function** (TOL)<br>**Spatial ability** (Mental Rotations Test; WAIS-III)	**Cortisol showed inverted-U-shaped associations with verbal recall** during pregnancy and postpartum and with **postpartum spatial ability**. **Prolactin** showed **linear and quadratic associations with recall and executive performance**. **Postpartum oestradiol and cortisol** were negatively associated with **attention**. Moderate hormone-cognition associations were observed (|r|=0.31–0.39).	Mood and other maternal characteristics assessed; examination of both linear and nonlinear models.
**Silber et al. (1990)**	**Longitudinal** and **case-control** study	**20 pregnant women** vs 20 non-pregnant controls; five assessments from late pregnancy to 12 months postpartum	Plasma **oxytocin**	**Visuo-spatial memory** (Benton test; Pattern Memory Test)<br>**Verbal memory** (Associate Learning Test; Late Recall Test)<br>**Attention** (Simple Reaction Time Test)	Peripartum cognitive performance was lower than at later postpartum assessments, but oxytocin concentrations were not significantly correlated with cognitive performance. Standardised effect sizes for the cognitive and oxytocin analyses were not reported.	Small sample; repeated assessments; no baseline data and no absolute level comparisons in cognitive results between groups.

Note. CANTAB = Cambridge Neuropsychological Test Automated Battery; CVLT = California Verbal Learning Test; JLO = Judgment of Line Orientation; SOP = Self-Ordered Pointing; SPWM = Spatial Working Memory; TOL = Tower of London; WAIS-III = Wechsler Adult Intelligence Scale, Third Edition

Table S2. Descriptive characteristics and main findings of the studies examining sleep health dimensions in relation to memory in pregnant women.

References	Study design	Sample size and age	Trimester	Sleep health dimensions	Type of memory	Main findings	Limitations
**Berndt et al. (2014)**	2-stage **longitudinal** and **case-control** study	**164 pregnant women** (M = 27.7 ± 4 y.o.; age range: 19-41 y.o.) vs. 58 non-pregnant controls	**2nd** and **3rd trimesters**: 15th-35th weeks (M = 22.2 ± 4.1)	Self-reported sleep quality/**Sleep satisfaction** (PSQI)	**Objective** memory:<br>• **False memories** (DRM paradigm)<br>• **Verbal working memory** (Digit span task, WAIS)	Significant positive correlations between **self-reported sleep difficulties** and percentage of **false alarms (r = 0.152), p < 0.05**.<br><br>Significant negative correlations between **self-reported sleep difficulties** and **verbal working memory (r = -0.156), p < 0.05**.	Cross-sectional findings; sample heterogeneity in gestational trimesters; low ecological validity of DRM paradigm.
**Hampson et al. (2015)**	2-stage **longitudinal** and **case-control** study	**28 pregnant women** (M = 29.96 ± 5.41 y.o.) vs 10 postpartum women vs 26 non-pregnant controls	**3rd trimester**: 34th-38th weeks	Awakening numbers/**Sleep efficiency** (one question)<br><br>**Sleep duration** (one question)	**Objective** memory:<br>• **Visuo-spatial working memory** (SPWM and SOP tasks)	Non-significant positive correlations between nighttime awakenings and SOP errors (r = 0.26) and SPWM errors (r = 0.01), p = ns.<br><br>Non-significant negative correlation between sleep duration and SOP errors (r = -0.10), p = ns.<br>Non-significant positive correlation between sleep duration and SPWM errors (r = 0.09), p = ns.	Cross-sectional findings; small sample size; non representative sample (Caucasian ethnicity, high education level, late pregnancy); limited and non-standardised sleep assessment.
**Nuckols et al. (2025)**	**Case-control** study	**84 pregnant women** (M = 31 ± 4 y.o.; age range: 23-44 y.o.) vs 73 non pregnant controls	**1st trimester** (10th-12th weeks of gestation)	**Sleep duration** (actigraphy)	**Objective** memory:<br>• **Verbal declarative memory** (RAVLT)	Non-significant negative association between sleep duration and retroactive interference (β = -1.1), p = ns.<br>Non-significant positive association between sleep duration and total learning (β = 0.8), p = ns.	Cross-sectional design; non representative sample (Caucasian ethnicity, high education level and high SES, early pregnancy).
**Zhang et al. (2021)**	**Cross-sectional** study	**1,818 pregnant women** (M = 31 ± 4.44 y.o.) (**Class 1**: good sleep quality, n = 882; **Class 2**: poor sleep efficiency, n = 190; **Class 3**: daily disturbances, n = 584; Class 4: poor sleep quality, n = 162)	**3rd trimester**: 38 ± 2.36 weeks	Self-reported sleep quality/**Sleep satisfaction** (PSQI) – Class 1 and 4<br><br>**Daytime sleepiness** (PSQI) – Class 3<br><br>**Sleep efficiency** (PSQI) – Class 2	**Subjective** memory:<br>• **Prospective and retrospective memory** (PRMQ)	Pregnant women with **daily disturbances** (Class 3 – B = 2.42) and **poor sleep quality** (Class 4 – B = 1.79) showed **more memory impairment** compared to women with good sleep quality (Class 1), **p < 0.001**.<br>No significant differences between women with poor sleep efficiency (Class 2 – B = 0.34) and women with good sleep quality (Class 1), p = ns.	Cross-sectional design; non representative sample (late pregnancy); reliance only on self-report measures; response bias risk.

References	Study design	Sample size and age	Trimester	Sleep health dimensions	Type of memory	Main findings	Limitations
**Zinke et al. (2021)**	**Case-control** study	**19 pregnant women** (M = 29.4 ± 1.2 y.o.; age range: 22-41 y.o.) vs 19 matched non-pregnant controls	**3rd trimester**: 29th-37th weeks (M = 33.2 ± 0.5)	Wake after sleep onset/**Sleep efficiency** (PSG)<br><br>**Sleep duration** (PSG)	**Objective** memory:<br>• **Procedural memory** (Finger-tapping task)<br>• **Visuo-spatial declarative memory** (Object-location memory task)<br>• **False memories**/Gist-based memories (DRM paradigm)	Non-significant positive correlations between sleep duration and<br>• percentage of procedural memory retention (r = 0.100), p = ns;<br>• percentage of visuo-spatial memory retention (r = 0.047), p = ns.<br>Non-significant negative correlation between sleep duration and<br>• number of gist words (r = -0.046), p = ns;<br>• list of words - adjusted recall (r = -0.295), p = ns.<br><br>Non-significant positive correlation between wake after sleep onset and<br>• percentage of procedural memory retention (r = 0.238), p = ns;<br>• percentage of visuo-spatial memory retention (r = 0.154), p = ns.<br>Non-significant negative correlation between wake after sleep onset and<br>• number of gist words (r = -0.448), p = ns;<br>• list of words - adjusted recall (r = -0.292), p = ns.	Cross-sectional findings; small sample size; non representative sample (late pregnancy).

Note. DRM = Deese-Roediger-McDermott; PRMQ = Prospective and Retrospective Memory Questionnaire; PSG = polysomnography; PSQI = Pittsburgh Sleep Quality Index; RAVLT = Rey Auditory Verbal Learning Task; SES = Socio-Economic Status; SOP = Self-Ordered Pointing; SPWM = Spatial Working Memory; WAIS = Wechsler Adult Intelligence Scale; WASO = Wake After Sleep Onset
